# In vivo Pharmacological Evaluations of Pilocarpine-Loaded Antioxidant-Functionalized Biodegradable Thermogels in Glaucomatous Rabbits

**DOI:** 10.1038/srep42344

**Published:** 2017-02-10

**Authors:** Shih-Feng Chou, Li-Jyuan Luo, Jui-Yang Lai

**Affiliations:** 1 Department of Mechanical Engineering, University of Texas at Tyler, Tyler TX, 75799, USA; 2 Department of Chemical and Materials Engineering, Chang Gung University, Taoyuan 33302, Taiwan, ROC; 3 Institute of Biochemical and Biomedical Engineering, Chang Gung University, Taoyuan 33302, Taiwan, ROC; 4 Biomedical Engineering Research Center, Chang Gung University, Taoyuan 33302, Taiwan, ROC; 5 Center for Tissue Engineering, Chang Gung Memorial Hospital, Taoyuan 33305, Taiwan, ROC; 6 Department of Ophthalmology, Chang Gung Memorial Hospital, Taoyuan 33305, Taiwan, ROC; 7 Department of Materials Engineering, Ming Chi University of Technology, New Taipei City 24301, Taiwan, ROC

## Abstract

To alleviate oxidative stress-induced ocular hypertension, grafting of antioxidant molecules to drug carriers enables a dual-function mechanism to effectively treat glaucomatous intraocular pressure (IOP) dysregulation. Providing potential application for intracameral administration of antiglaucoma medications, this study, for the first time, aims to examine *in vivo* pharmacological efficacy of pilocarpine-loaded antioxidant-functionalized biodegradable thermogels in glaucomatous rabbits. A series of gallic acid (GA)-grafted gelatin-*g*-poly(*N*-isopropylacrylamide) (GN) polymers were synthesized via redox reactions at 20–50 °C. Our results showed that raising redox radical initiation reaction temperature maximizes GA grafting level, antioxidant activity, and water content at 40 °C. Meanwhile, increase in overall hydrophilicity of GNGA carriers leads to fast polymer degradation and early pilocarpine depletion *in vivo*, which is disadvantageous to offer necessary pharmacological performance at prolonged time. By contrast, sustained therapeutic drug concentrations in aqueous humor can be achieved for long-term (i.e., 28 days) protection against corneal aberration and retinal injury after pilocarpine delivery using dual-function optimized carriers synthesized at 30 °C. The GA-functionalized injectable hydrogels are also found to contribute significantly to enhancement of retinal antioxidant defense system and preservation of histological structure and electrophysiological function, thereby supporting the benefits of drug-containing antioxidant biodegradable thermogels to prevent glaucoma development.

Clinically, glaucoma is diagnosed as a progressive and irreversible ocular disease that eventually leads to blindness. It is estimated that 76.0 million and 111.8 million people will suffer from glaucoma in 2020 and 2040, respectively, according to a regression model^1^. Although the origin of the disease is still unknown, research has showed that abnormal elevation of intraocular pressure (IOP) is a high risk factor for the development of glaucoma^2^. In particular, ocular hypertension triggers excavation of optic nerve head, which further disrupts axonal transport of retinal ganglion cells and results in cell degeneration and death through an apoptotic mechanism^3^. Other investigators have correlated inner plexiform layer damage with glaucoma due to IOP elevation^4^. These mechanisms illustrate the significant role of ocular hypertension in the regulation of retinal tissue injury during disease progression where current treatment modalities in glaucomatous patients have been focused on the prevention of abnormal elevation of IOP.

Among all clinical procedures to lower IOP in treatments against glaucoma, intracameral injection of biodegradable and biocompatible temperature-triggered polymer carriers showed pharmacological potentials as an alternative method to strategically administer pilocarpine in glaucomatous eyes^5^. Additionally, in order to alleviate oxidative stress that causes ocular hypertension, grafting of antioxidant molecules to the drug carriers enabled a dual-function mechanism to effectively treat glaucomatous IOP dysregulation^6^. Recently, the effects of redox reaction time on the synthesis of antioxidant-functionalized biodegradable *in situ* gelling polymers for intracameral pilocarpine administration were examined^7^. Results from the short-term study showed that reaction time significantly affected the grafting amount of antioxidant on carrier materials, which determined polymer degradability to further modulate drug release and therapeutic performance. As shown in Fig. 1, the molecular functionalization of biodegradable thermogels with antioxidants is achieved by radical reaction in the presence of hydrogen peroxide/ascorbic acid redox pair as an initiator. At different redox radical initiation reaction temperatures, the attack of the residues in the protein side chains of the biodegradable thermogels can produce varying amounts of radical species that subsequently react with antioxidant molecules to form the resultant polymeric carriers. Therefore, similar to the role of reaction duration in functionalization of vehicle materials with antioxidants, redox radical initiation reaction temperature may be able to maximize grafting amount of antioxidant molecules on antiglaucoma polymeric drug delivery systems. In particular, current study shows that redox radical initiation reaction temperature also significantly affects physicochemical properties of the antioxidant-functionalized injectable hydrogels, resulting in differences of pharmacological efficacies on *in vivo* controlled pilocarpine delivery for treatment of glaucoma-induced corneal and retinal injuries over 28 days.

Apart from our previous works, the current study focuses on pharmacological effects of intracameral drug carriers, synthesized from chemical grafting of antioxidant gallic acid (GA) onto biodegradable gelatin and thermo-responsive poly(*N*-isopropylacrylamide) (GN) copolymer at different redox radical initiation reaction temperatures, on glaucomatous tissues. We hypothesize that increasing redox radical initiation reaction temperature increases the grafting amount of GA to an optimal level, thereby providing antioxidant benefits while accommodating controlled release of pilocarpine from carrier materials *in vivo*. Specifically, total antioxidant activity, radical scavenging ability, water content, phase transition temperature, degradability, and drug encapsulation efficiency of GNGA samples suggest a dependence on redox radical initiation reaction temperatures (20–50 °C). The antioxidant functions of GNGA carriers show ability to alleviate oxidative stress in an *in vitro* model of hydrogen peroxide-induced lens epithelial cell injury. Furthermore, carrier materials are biocompatible in the anterior chamber of rabbit eyes. The bioavailability of the delivered ocular drug from GNGA carriers was separately studied using a rabbit model of experimental glaucoma. Follow-up IOP measurements as well as pharmacological responses at postoperative week 4, including corneal topography, electroretinogram, and retinal histology and antioxidant defense system activity were investigated on animals receiving intracameral pilocarpine/polymer injections. Our findings contribute significantly to the understanding of therapeutic efficacy of the antioxidant-functionalized injectable hydrogels and the role of redox radical initiation reaction temperature in developing carrier biomaterials for the treatment of glaucomatous injury.

## Results

### Characterization studies

Multifunctional intracameral GNGA carriers^6^,^7^, synthesized from various redox radical initiation reaction temperatures, exhibit different physicochemical properties (Table 1). Total antioxidant activities of GNGA carriers determined by phosphomolybdenum assays significantly increase from 0.1 ± 0.04 mg/g polymer (T20 groups) to 1.5 ± 0.07 mg/g polymer (T40 groups) (*P* < 0.05), followed by a slight reduction to 1.3 ± 0.11 mg/g polymer (T50 groups) when increasing redox radical initiation reaction temperature from 20 to 50 °C. A similar trend is also observed on the scavenging ability of GNGA materials against 2,2′-diphenyl-1-picrylhydrazyl (DPPH) radicals, suggesting that T40 groups receive the highest grafting amount of GA. Due to the hydrophilic nature of the grafted antioxidant molecules, the T40 samples exhibit a relatively high water content of 78.1 ± 1.8%. Increasing hydrophilicity in GNGA materials increases phase transition temperature as a result of higher required energy to disrupt polymer-water interaction^8^. Water content also determines the degradability of GNGA where T40 groups achieve a 72.6 ± 1.3% weight loss after 28 days in physiological condition. On the other hand, pilocarpine encapsulation efficiency increases with increasing redox radical initiation reaction temperature from 20 to 50 °C, indicating the dependence of drug encapsulation level of functionalized GNGA on the grafting amount of GA. High drug encapsulation efficiency combined with fast degradation rate from T40 groups result in over 95% of pilocarpine cumulative release *in vitro* after 2 days (Figure S1). In summary, characterizations of GNGA carriers obtained at various redox radical initiation reaction temperatures show that T40 samples exhibit the highest degree of antioxidant grafting and strongest GA molecule-mediated change in carrier properties among all studied groups.

### *In vitro* antioxidant activity studies

**Figure 2:**
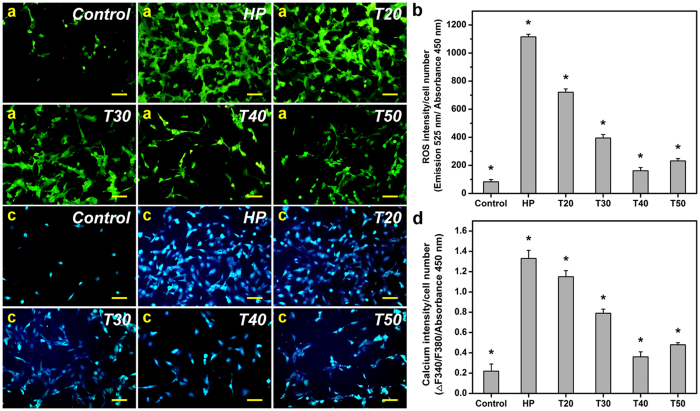
*In vitro* antioxidant activity studies. Effect of polymer carrier materials on H_2_O_2_-induced intracellular (**a**,**b**) ROS and (**c**,**d**) calcium. (**a**,**c**) Representative fluorescent images of the HLE-B3 cells after incubation with various GNGA samples T20, T30, T40, and T50 for 24 h and further exposure to H_2_O_2_ for 24 h. The cells exposed to 0 (Control group) or 200 (HP group) μM H_2_O_2_ for 24 h following 24 h of incubation in the absence of the polymers are used for comparison. Scale bars: 30 μm. (**b**,**d**) Measurement of antioxidant activity against oxidative stress. Intracellular levels of ROS and calcium are respectively measured by the fluorescence intensity of DCFH-DA and Fura-2, AM with a microplate reader. The fluorescence intensity at 525 nm is used to determine the ROS production. The fluorescence ratio (F340/F380) calculated based on the emissions at the excitation wavelength of 340 and 380 nm is used to determine the calcium concentration. Data are normalized to the total cell number determined by WST-1 (absorbance of 450 nm). Values are mean ± standard deviation (*n* = 3). *P < 0.05 vs all groups.

Glaucomatous eyes are often characterized with a high level of oxidative stress that can lead to cell death^9^,^10^. Given that GNGA materials exhibit excellent free radical scavenging abilities due to grafting of GA molecules, *in vitro* oxidative challenges are employed to evaluate their antioxidant capacities. As shown in Figure S2, cell morphology and viability depend on the grafting amount of antioxidant molecules in GNGA biomaterials (also see the Supplementary Information). High intensities of green fluorescence in human lens epithelial (HLE-B3) cell line cultures (HP groups) subjected to hydrogen peroxide-induced oxidative environment indicate the generation of large amount of reactive oxygen species (ROS) (Figs 2a and S3a). In addition, the intensity of green fluorescence decreases from pretreatment of GNGA materials on HLE-B3 cells, suggesting a protection from polymeric carrier against ROS production. Quantitative analysis of the intensity at wavelength of 525 nm shows the lowest ROS generation from T40 groups followed by T50 groups, T30 groups, and T20 groups, respectively (Fig. 2b). Of equal importance, intracellular overload of calcium is critical to HLE-B3 cell survival where a high level of intracellular calcium is often related to cell death caused by oxidative stress^11^,^12^. Strong intensities of blue fluorescence refer to high intracellular calcium content in HLE-B3 cell cultures (HP groups) after oxidative challenges (Figs 2c and S3b). Similar to the findings in ROS assay, intracellular calcium level is reduced from pretreatment of cells using GNGA carriers. Quantitative analysis of the intensity at wavelength of 550 nm reveals the lowest intracellular calcium content from T40 groups, which is close to that of the control groups (Fig. 2d). Intracellular overload of calcium then increases in T50 groups followed by T30 and T20, respectively. Both observations are in accordance with previous physicochemical findings in total antioxidant activity and free radical scavenging ability of GNGA carriers (Table 1). In general, we demonstrate that GNGA materials protect lens epithelial cells from oxidative environment where the antioxidative effect strongly depends on redox radical initiation reaction temperature-mediated degree of GA grafting.

### *In vivo* biocompatibility studies

**Figure 3:**
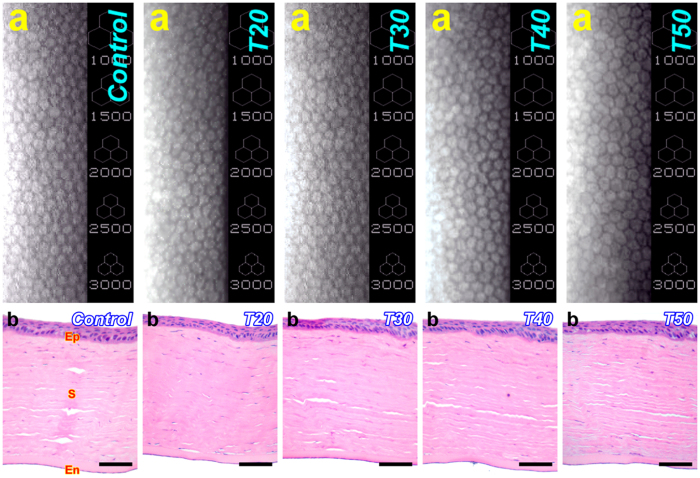
*In vivo* biocompatibility studies. Representative (**a**) specular microscopic images of rabbit corneal endothelium and (**b**) corneal histology after 28 days of intracameral injection with various GNGA samples T20, T30, T40, and T50. Control group: sham operation (no material). Sections are stained with H&E. Ep: epithelium; S: stroma; En: endothelium. Scale bars: 100 μm.

Injections of GNGA carriers to the ocular anterior chamber present a viable solution for the treatment of glaucoma patients. Here, we examine the *in vivo* biocompatibility of corneal endothelium, a flattened layer with hexagonal cell profiles stacked in a honeycomb pattern^13^, with GNGA materials for 28 days using specular microscopy (Fig. 3a). Endothelial tissues from all studied groups display their characteristic cellular morphology with hexagonal profiles. In addition, quantitative analysis on endothelial cell density suggests no significant difference between the control, T20, T30, T40, and T50 groups (*P* > 0.05) (Figure S4a). Our observations confirm the *in vivo* biocompatibility of GNGA carriers with corneal endothelium. Histological examinations using hematoxylin and eosin (H&E) staining on rabbit cornea reveal a multilayered tissue structure comprising an outer epithelium, a central stroma with keratocytes and orthogonally arranged collagen lamellae, and an inner endothelium (Fig. 3b). After GNGA injection for 28 days, no inflammatory response and/or abnormal neovascularization in corneal tissues exposed to T20, T30, T40, and T50 samples are observed. Furthermore, the concentrations of interleukin-6 (IL-6) in aqueous humor did not show a significant difference between the control, T20, T30, T40, and T50 groups (*P* > 0.05), which indicates that the intracamerally injected GNGA samples does not promote inflammation in the anterior chamber (Figure S4b). Overall, *in vivo* ocular biocompatibility studies provide important information to support the safety of intracamerally injected GNGA materials.

### *In vivo* drug release studies

**Figure 4:**
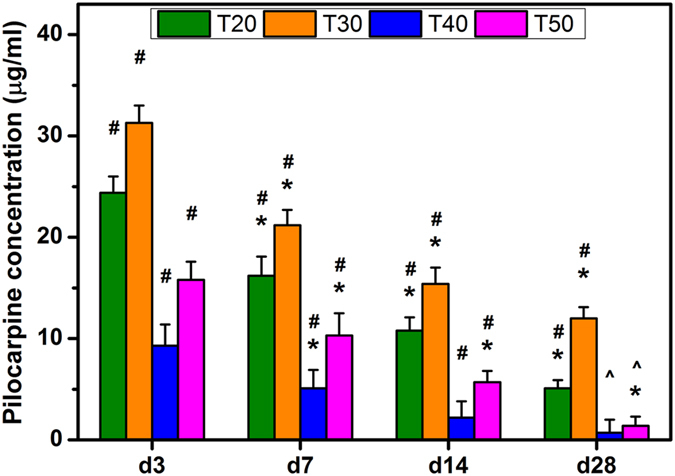
*In vivo* drug release studies. Time-course of the concentration of pilocarpine released from various GNGA samples T20, T30, T40, and T50 in aqueous humor. An asterisk indicates statistically significant differences (*P < 0.05; *n* = 3) for the mean value of the pilocarpine concentration compared to the value at the previous time point. ^#^*P* < 0.05 vs all groups (compared only within each time point group). Follow-up time point: day (d).

After successful induction of experimental glaucoma by using α-chymotrypsin^5^, mixtures of pilocarpine and GNGA carriers obtained at various redox radical initiation reaction temperatures from 20 to 50 °C were delivered to the anterior chamber of rabbit eyes via injection. *In vivo* pilocarpine concentrations released from GNGA materials at various time points up to 28 days show that drug concentrations in aqueous humor gradually decrease over time where the order of ranking in concentration at each time point follows T30 > T20 > T50 > T40 (Fig. 4). During the follow-up, the low concentration of released drug in the T40 groups is attributed to burst release characteristic of the carriers (Figure S1) combined with fluid circulation within the anterior chamber. By contrast, T30 groups exhibit high pilocarpine concentration above therapeutic level (10 μg/ml^5^) up to 28 days. These findings are associated with overall hydrophilicity of GNGA materials and their resistance to degradation, which are further determined by degree of GA grafting. In particular, *in vivo* pilocarpine release is critical in regulation of IOP since cholinergic drug tightens iris/ciliary body resulting in pupillary constriction and trabecular meshwork enlargement that allow excess aqueous humor to drain away from anterior chamber^14^. As a result, IOP profiles of rabbit eyes receiving drug-containing GNGA injections decrease to near baseline value followed by different increasing rates at later time points depending on their corresponding *in vivo* drug release behaviors (Figure S5). Specifically, T40 groups are the most hydrophilic drug carriers among all studied groups due to high grafting amount of GA that yields the fastest polymer degradation rate (Table 1). Degradation-dependent release kinetics in GNGA carriers leads to early depletion of drug *in vivo* (Fig. 4), and therefore, IOP profiles of GNGA carriers display different decrease and increase rates in 28 days. In particular, T30 groups show a better overall IOP reduction where the differences of IOP values between treated eyes and normal eyes are within 5 mmHg from 1 to 28 days. Our results correlate *in vivo* drug concentrations with IOP profiles where pharmacological benefits strongly depend on the delivery performance of GNGA carriers obtained at different redox radical initiation reaction temperatures.

### Corneal topography measurements

**Figure 5:**
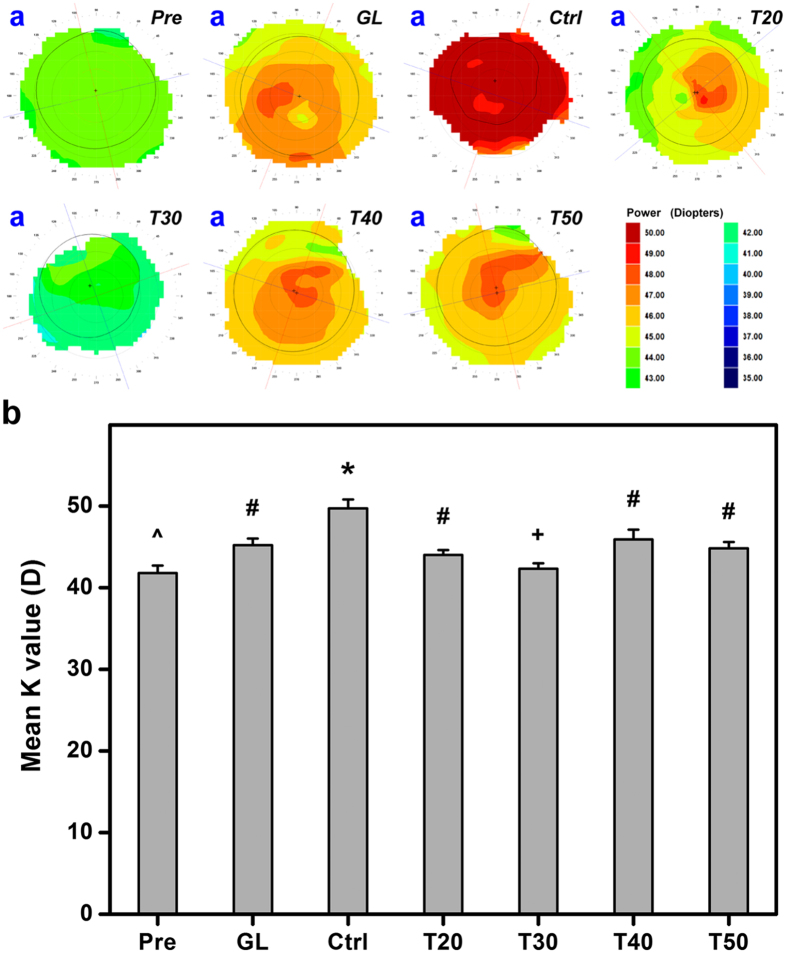
Corneal topography measurements. (**a**) Representative corneal topographic maps of rabbit eyes at preoperation (Pre) and those with experimentally induced glaucoma (GL) 28 days after intracameral injection of pilocarpine-containing GNGA polymer solutions (T20, T30, T40, and T50). Glaucomatous animals receiving no polymer and drug serve as control groups (Ctrl). (**b**) Topography measurements of corneal curvature (mean K). Values are mean ± standard deviation (*n* = 6). *P < 0.05 vs all groups; ^^^*P* < 0.05 vs all groups, except T30; ^+^*P* < 0.05 vs all groups, except Pre; ^#^*P* < 0.05 vs Pre, Ctrl, and T30 groups.

Corneal topographic map of normal rabbit eyes (Pre groups) shows a relatively low level of corneal aberration indicating by the green zone (Fig. 5a). After experimental induction of glaucoma, corneal topography in response to IOP elevation displays an increased level of wavefront aberrations in the anterior corneal surface (GL groups). At 28 days postoperatively, a red zone in topographic map is found in Ctrl groups, suggesting a steeper curvature with a higher refractive power occurring due to disease progression. By contrast, intracameral injections of various drug-containing GNGA carriers reduce corneal aberration at levels that depend on their corresponding *in vivo* drug release profiles and IOP lowering effects. For example, T30 groups receive the lowest level of aberration, which is close to preoperative eyes (Figs 4 and S5). In addition, T40 and T50 groups exhibit some levels of aberration, mostly attributed to increased IOP associated with the depletion of drug at prolonged follow-up time. These observations suggest a strong correlation between IOP elevation and variation in corneal contour, which implies the importance of maintaining pilocarpine *in vivo* to a desirable therapeutic level. Furthermore, the mean keratometric (K) value (i.e., mean simulated keratometry), recorded from corneal topography measurements, of normal rabbits from Pre (41.8 ± 0.9 D) groups is significantly lower than that of glaucomatous rabbits from Ctrl (49.7 ± 1.1 D) groups (*P* < 0.05) (Fig. 5b). At 28 days postoperatively, there is no significant difference in mean K value between the GL (45.2 ± 0.8 D), T20 (44.0 ± 0.6 D), T40 (45.9 ± 1.2 D), and T50 (44.8 ± 0.8 D) groups (*P* > 0.05). The low mean K value in T30 (42.3 ± 0.7 D) groups, which is close to the preoperative condition, also strengthens the link between IOP and corneal curvature. In general, our findings suggest that corneal topographies and their mean K values depend on the pharmacological efficacy of intracameral drug-containing GNGA injections.

### Electroretinogram measurements

**Figure 6:**
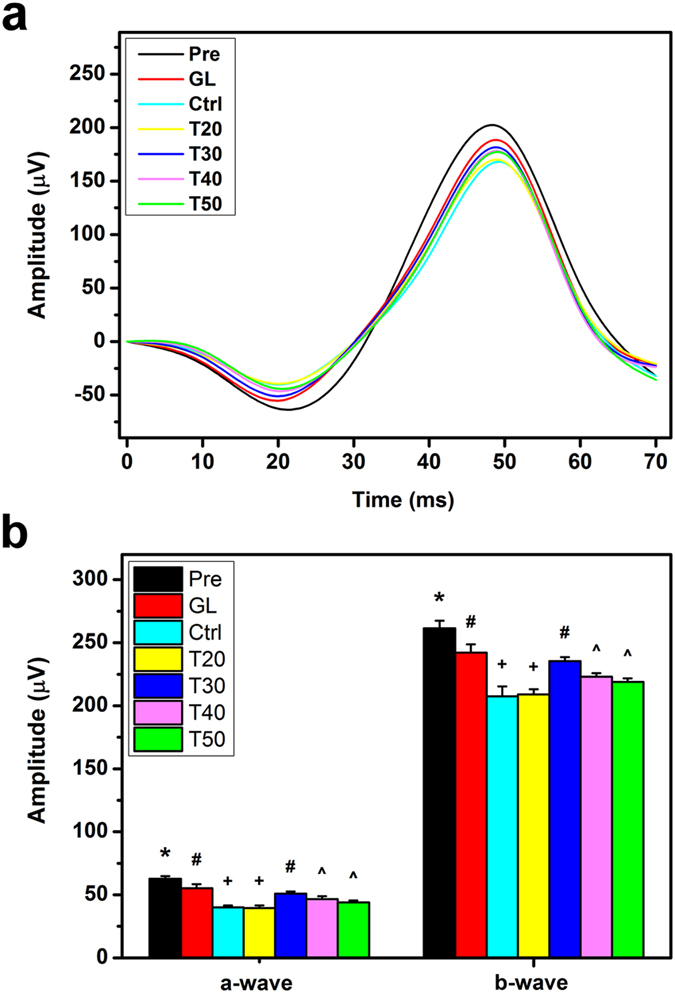
Electroretinogram measurements. (**a**) Representative ERG recordings of rabbit eyes at preoperation (Pre) and those with experimentally induced glaucoma (GL) 28 days after intracameral injection of pilocarpine-containing GNGA polymer solutions (T20, T30, T40, and T50). Glaucomatous animals receiving no polymer and drug serve as control groups (Ctrl). (**b**) Quantification of a- and b-wave amplitudes. Values are mean ± standard deviation (*n* = 6). *P < 0.05 vs all groups; ^#^*P* < 0.05 vs Pre, Ctrl, T20, T40, and T50 groups; ^^^*P* < 0.05 vs Pre, GL, Ctrl, T20, and T30 groups; ^+^*P* < 0.05 vs Pre, GL, T30, T40, and T50 groups.

During visual electrophysiological tests, electroretinogram (ERG) patterns are often used for glaucoma diagnosis where the reduction in waveform amplitude is associated with ocular hypertension in glaucoma patients^15^. Typical ERG waveform spectra are characterized with a small and negative a-wave originated from photoreceptors followed by a large and positive b-wave produced from bipolar and Müller cells in retinal tissue^16^. Our results show that ERG profiles of healthy rabbit eyes, glaucomatous eyes, and those treated with drug-containing GNGA injections after 28 days exhibit variations in waveform amplitude (Fig. 6a). In particular, waveform configurations of glaucomatous eyes and those treated with various GNGA carriers are generally similar to the preoperative eyes. However, differences in waveform amplitudes within T20-T50 groups suggest variations in corresponding electrophysiological state of the retina at 28 days of follow-up. Quantitative analysis of ERG waveform amplitudes suggests that a-wave and b-wave amplitudes are 62.8 ± 2.1 μV and 261.4 ± 6.1 μV in healthy eyes (Pre groups), respectively (Fig. 6b). These values are in accordance with the reported values in literature^17^. In addition, the a-wave and b-wave amplitudes in Ctrl groups significantly decrease to 40.1 ± 1.5 μV and 207.5 ± 7.8 μV, respectively (*P* < 0.05), suggesting a profound loss of electrophysiological function of retina in glaucomatous eyes. By contrast, waveform amplitudes of a-wave and b-wave significantly increase after injections of drug-containing GNGA samples from T30, T40, and T50 groups at postoperative day 28 as compared to the Ctrl groups (*P* < 0.05). Among all studied groups, T30 groups show the highest increases in waveform amplitudes of a-wave and b-wave closest to those of the GL groups, indicating that the carrier material has the strongest potential in preventing cell death in retinal tissue from glaucoma due to high concentration of released pilocarpine *in vivo* (Fig. 4) that results in the decrease of IOP (Figure S5). Moreover, the grafting amount of GA molecules in T30 groups may be sufficient to provide a considerable level of antioxidant capacity to defend against ROS attack in the retinal cells. Overall, ERG patterns reveal decreases in waveform amplitudes in glaucomatous eyes where current results demonstrate that drug-containing GNGA injections (T30 groups) prevent the loss of retinal electrophysiological functions.

### Retinal histological examinations

**Figure 7:**
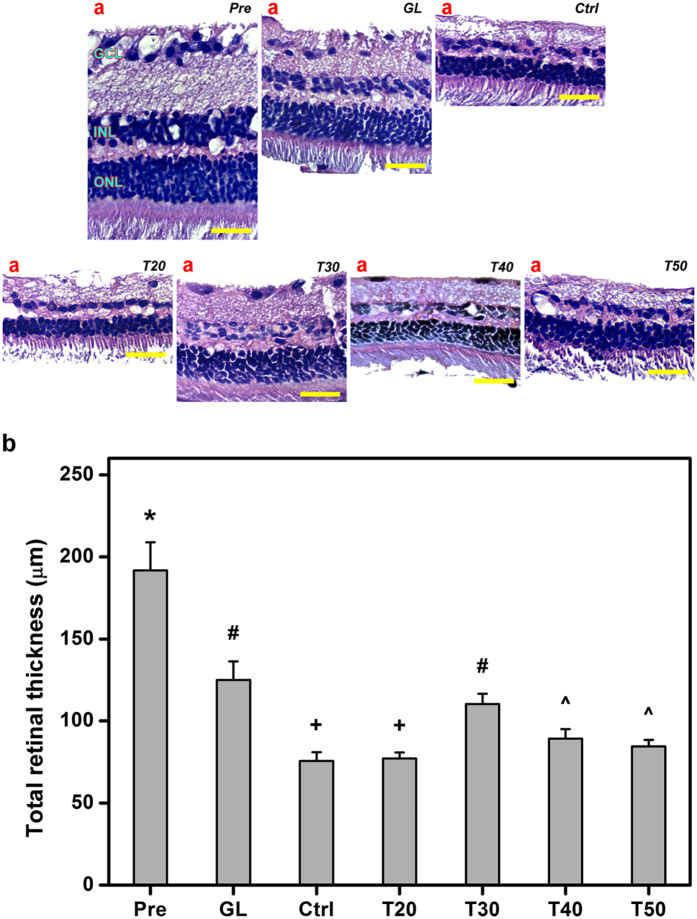
Retinal histological examinations. (**a**) Representative histological images of retina at preoperation (Pre) and those with experimentally induced glaucoma (GL) 28 days after intracameral injection of pilocarpine-containing GNGA polymer solutions (T20, T30, T40, and T50). Glaucomatous animals receiving no polymer and drug serve as control groups (Ctrl). Sections are stained with H&E. GCL: ganglion cell layer; INL: inner nuclear layer; ONL: outer nuclear layer. Scale bars: 50 μm. (**b**) Histologic measurements of total retinal thickness. Values are mean ± standard deviation (*n* = 6). *P < 0.05 vs all groups; ^#^*P* < 0.05 vs Pre, Ctrl, T20, T40, and T50 groups; ^^^*P* < 0.05 vs Pre, GL, Ctrl, T20, and T30 groups; ^+^*P* < 0.05 vs Pre, GL, T30, T40, and T50 groups.

The structure of normal retina (Pre groups) consists of stacked ganglion cell layer (GCL), inner nuclear layer (INL), and outer nuclear layer (ONL) where thickness reduction and ratio in numbers of cells between INL and ONL are evidence of glaucoma progression^18^. Histological results show that the retinal tissues suffer from a significant loss of GCL and thinning of INL and ONL in glaucomatous eyes (both GL and Ctrl groups) as compared to those from preoperative eyes (Pre groups) (Fig. 7a). In addition, the rabbits receiving drug-containing GNGA injections from T20, T30, T40, and T50 groups show different retinal histologic features at 28 days postoperatively. In particular, T30 groups have a retinal structure closest to that of glaucomatous eyes, suggesting that intraocular delivery of pilocarpine using antioxidant-containing carrier material may be able to mitigate the irreversible degeneration of retinal neurosensorial tissue. Results of quantitative analysis show that total retinal thickness in healthy rabbit eyes is 191.8 ± 17.1 μm followed by a significantly decrease to 125.1 ± 11.2 μm in glaucomatous eyes (*P* < 0.05) (Fig. 7b). The 35% reduction in total retinal thickness in current study is similar to the reported value of 43% decrease in a rat model of IOP elevation^19^. At 28 days postoperatively, the retinal thicknesses in the Ctrl, T20, T30, T40, and T50 groups are 75.6 ± 5.4 μm, 77.2 ± 3.6 μm, 110.4 ± 6.3 μm, 89.3 ± 5.7 μm, and 84.5 ± 4.0 μm, respectively. Given that the decreases in total retinal thickness are associated with the decreases in waveform amplitudes in ERG (Fig. 6), T20 groups do not have the ability to maintain retinal function. Our data clearly demonstrate that further loss and thinning of the retinal cellular layers are noticeable in glaucomatous eyes without any drug/polymer treatment, where T30 groups can better prevent both conditions attributing to dual pharmacological actions of released pilocarpine and antioxidative effects of carrier material.

### Biochemical assays

**Figure 8:**
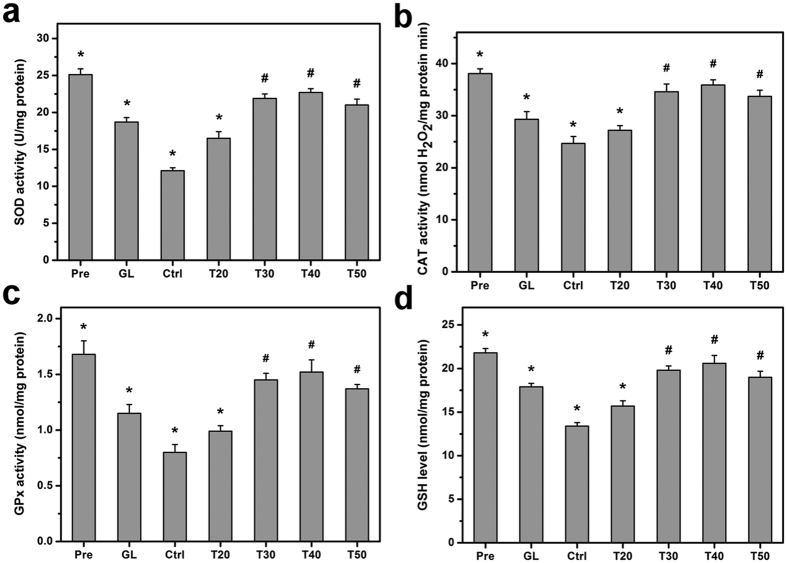
Biochemical assays. (**a**) SOD activity, (**b**) CAT activity, (**c**) GPx activity, and (**d**) GSH level in the retina of rabbit eyes at preoperation (Pre) and those with experimentally induced glaucoma (GL) 28 days after intracameral injection of pilocarpine-containing GNGA polymer solutions (T20, T30, T40, and T50). Glaucomatous animals receiving no polymer and drug serve as control groups (Ctrl). Values are mean ± standard deviation (*n* = 6). *P < 0.05 vs all groups; ^#^*P* < 0.05 vs Pre, GL, Ctrl, and T20 groups.

Glaucoma is an optic neuropathy involving the death of retinal ganglion cells attacked and damaged by free radicals^20^. These free radicals oxidize polyunsaturated fatty acids in a tissue environment that has high oxygen content combined with direct exposure to light resulting in the generation of excessive oxidative stress^21^. Glutathione (GSH) and several antioxidant enzymes, including superoxide dismutase (SOD), catalase (CAT), and glutathione peroxidase (GPx), are able to maintain a strong retinal antioxidant defense against oxidative damage^22^. Results show that SOD, CAT, and GPx levels in preoperative eyes are 25.1 ± 0.8 U/mg protein, 38.1 ± 0.9 nmol H_2_O_2_/mg protein min, and 1.68 ± 0.12 nmol/mg protein, respectively (Fig. 8a–c). These findings are in agreement with the reported literature values^22^. In addition, SOD, CAT, and GPx level decrease significantly to 12.1 ± 0.4 U/mg protein, 24.7 ± 1.3 nmol H_2_O_2_/mg protein min, and 0.80 ± 0.07 nmol/mg protein, respectively (*P* < 0.05), in glaucomatous eyes without treatments after 28 days (Ctrl groups), suggesting the effect of oxidative damage. By contrast, injections of drug-containing GNGA samples mitigate the decrease in SOD, CAT, and GPx activities where the order of ranking is T40 > T30 > T50 > T20 groups. In particular, T40 groups with the highest degree of antioxidant grafting have a retinal antioxidant defense system activity closest to that of preoperative eyes. Furthermore, GSH is an important antioxidant in tissues where its reduced form may be used by GPx and serves as a detoxifier against free radicals and oxidative stress^23^. Our results show that GSH levels follow the same trend as SOD, CAT, and GPx activities as described above (Fig. 8d). It is interesting to note that although the carriers from T30 groups exhibit a significantly lower grafting amount of GA than their T50 counterparts (*P* < 0.05), the concentration of released pilocarpine can effectively regulate the IOP to near baseline value during the follow-up, thereby alleviating ocular hypertension-induced retinal oxidative stress^22^. Furthermore, the results of ERG measurements, retinal histological examinations, and biochemical assays showed that the mixtures of pilocarpine and GNGA (T50 groups) have a much higher therapeutic efficacy in preventing retinal damage of glaucomatous rabbits than GNGA alone (T50(w/o) groups) at postoperative 7 days, again suggesting the importance of dual pharmacological actions of released pilocarpine and antioxidative effects of carrier material. Overall, we demonstrate that for the animals receiving the mixtures of pilocarpine and GNGA carriers obtained at various redox radical initiation reaction temperatures, both IOP control and antioxidant defense play essential roles in retinal antioxidant activity level for maintenance of a healthy metabolism against glaucoma development.

## Discussion

The use of antioxidant-functionalized biodegradable *in situ* gelling carriers for intracameral pilocarpine administration improved the therapeutic efficacy of antiglaucoma medication in disease treatment^6^. These multifunctional GNGA polymeric carriers intentionally designed for glaucoma treatment exhibited different *in vitro* drug release behaviors for 14 days while release mechanisms were associated with degradation of carrier materials using redox reaction time-mediated grafting amount of antioxidant molecule^7^. In this study, we found that redox radical initiation reaction temperature is equally important in determining physicochemical properties of GNGA carriers, leading to variations of *in vivo* drug release that corresponded to levels of pharmacological performance for treating glaucoma-induced corneal and retinal injuries over 28 days. In the range of 20–50 °C, total antioxidant activity and free radical scavenging ability of GNGA materials increase with increasing redox radical initiation reaction temperature to 40 °C followed by a slight decrease at 50 °C, suggesting the effect of redox reaction conditions on grafting amount of antioxidant molecule. In a study, radical polymerization of 6-*O*-vinyladipoyl d-glucose using ascorbic acid and hydrogen peroxide showed a dependence of molecular interactions on redox radical initiation reaction temperature between 25 and 55 °C^24^. A similar effect was reported using either ferrous ammonium sulfate-potassium persulfate or Fenton’s reagent (Fe^2+^-H_2_O_2_) as the redox initiator on graft copolymerization of vinyl monomers onto cellulose^25^. In particular, Fenton’s reagent had an optimum temperature in affording maximum grafting at 30 °C whereas the maximum grafting in the presence of ferrous ammonium sulfate-potassium persulfate occurred at 55 °C. Increasing redox radical initiation reaction temperature accelerates the diffusion of monomer and decomposition of hydrogen peroxide, thereby favoring the grafting reaction. Nevertheless, when the reaction temperature is raised above the optimum temperature for maximum grafting, the grafting ratio is decreased due to poor selectivity and various hydrogen abstraction^25^. A typical redox reaction consists of several grafting mechanisms, including generation of free radicals, chain initiation, chain propagation, chain termination, and homopolymer formation^26^. Among all these grafting mechanisms, chain propagation (chain transfer reactions) and chain termination appear to mainly rely on redox radical initiation reaction temperature. For example, increasing reaction temperature accelerates chain transfer reactions and chain termination reactions in graft copolymerization of eucalyptus lignosulfonate calcium from hardwood and acrylic acid, and therefore decreases percent grafting at a higher temperature above maximum grafting ratio^27^. Consistent with these literature reports, our results of antioxidant activity studies show that during the synthesis of GNGA materials, the optimum temperature for maximum grafting is 40 °C while continuing increasing redox radical initiation reaction temperature slightly decreases grafting amount of GA.

Based on the characterization results in this study, it may be straightforward to determine that T40 groups receive the best performance in pharmacological efficacy to treat glaucoma due to the highest grafting amount of antioxidant molecule. However, increasing GA grafting enhances the ability of carrier materials to encapsulate hydrophilic pilocarpine attributed to the increase in overall GNGA hydrophilicity. This effect also facilitates swelling of GNGA materials and leads to a faster degradation rate, which plays an important role in drug release^28^. Here, the T40 groups receive relatively high weight loss that depletes drug concentration to the level of less than 10 μg/ml *in vivo* within 3 days. On the other hand, T30 groups exhibit the highest *in vivo* drug concentration among all studied groups over 28 days, suggesting the dependence of controlled-release behavior of GNGA carriers on polymer degradation^29^. Others reported a 34% cumulative release of paclitaxel from injectable thermosensitive hydrogels consisted of poly(ε-caprolactone)–poly(ethylene glycol)–poly(ε-caprolactone) amphiphilic copolymers after 6 weeks of incubation in phosphate-buffered saline at 37 °C^30^. Interestingly, incorporation of hydrophilic pendant cyclic ether groups (i.e., 1,4,8-trioxa[4.6]spiro-9-undecanone) in the poly(ε-caprolactone) block increased polymer degradation rate, thereby increasing paclitaxel cumulative release to 64%. In another study, an increased salicylic acid release from glyceryl monooleate (a polar lipid)/water system with an enlarged hydrophilic domain was found^31^. Similarly, thermosensitive poly(ethylene glycol)-grafted chitosan hydrogels showed that the release of bovine serum albumin depended on the material degradation rate modulated by the grafting ratio of poly(ethylene glycol)^32^. These examples demonstrate the importance of degradation-mediated drug release from hydrogel carriers where the overall hydrophilicity determines the degradation rate of the hydrogel materials. Our results show that T30 groups receive a moderate degree of GA grafting that modifies the hydrophilic nature of delivery carriers to achieve sustained release of pilocarpine over 28 days. The effects of appropriate pharmacological dose and antioxidant activity from carrier materials in T30 groups suggests a potential application in glaucoma treatment.

Glaucoma development and progression is often characterized with a high level of intraocular oxidative stress and pressure that leads to alteration in corneal topography and damage to retinal structure and function. Our data clearly demonstrate the attenuation of the anterior corneal aberration in response to IOP reduction in the T30 groups, suggesting a strong correlation between antiglaucoma efficacy and effective therapeutic pilocarpine level *in vivo*. On the other hand, in clinical trials, glaucoma patients typically receive antioxidant supplements in order to lower the abnormal oxidative stress levels. For example, propolis (a honeybee product that exhibits antioxidative ability) solution was administered intraperitoneally at 48 h, 24 h and 60 min before and at 6 h sequentially after experimental induction of retinal damage using *N*-methyl-d-aspartate (NMDA) in a mouse model^33^. The results indicated that propolis mitigated the reduction of retinal ganglion cells as well as the thinning of the inner plexiform layer. Similarly, edaravone (a free radical scavenger) was intravenously injected to protect against NMDA-induced retinal injury where increasing concentration of edaravone significantly inhibited ganglion cell loss^34^. Others have shown that injections of highly concentrated antioxidant solutions could prevent NMDA-induced retinal damage or light-induced photoreceptor degeneration^35^,^36^. In addition, topical delivery of Coenzyme Q10 using eye drop formulations was reported to protect retina from UV-induced stress^37^. These studies highlight that a wide variety of antioxidants used for retinal cytoprotection can be given via different administration routes. While it is obvious that administration of these antioxidants can mitigate retinal tissue damage, it is unclear whether the functionalization of antioxidant molecules onto the polymeric drug carriers can influence their antioxidative retinal cytoprotection in a glaucomatous rabbit model. Herein, our results suggest that intraocular injection of pilocarpine-containing GNGA materials provides sufficient protection against retinal damage after experimentally induced glaucoma in animals. Of equal importance, we show that *in vivo* pharmacological performance and therapeutic efficacy depend on grafting amount of GA-mediated hydrophilicity of the carrier materials, which is further determined by redox radical initiation reaction temperature. This effect is demonstrated in retinal histological examinations and ERG measurements where retinal tissue structure and electrophysiological function from T30 groups are the closest to those of glaucoma eyes (GL groups), which are much improved from eyes without treatment (Ctrl groups). Furthermore, our findings on biochemical assays support that T30 groups achieve the second highest SOD, CAT, GPx, and GSH levels behind T40 groups among all studied groups. While pilocarpine level and antioxidant capacity *in vivo* may complicate the mechanism in the change of these enzyme activities, we demonstrate that intraocular drug/polymer injections (T30 groups) provides 1.4–1.8 fold of increase in retinal antioxidant defense system activities as compared to those without treatment (Ctrl groups). The present work suggests that antioxidant-functionalized GNGA carrier materials may help to limit glaucomatous disease progression associated with oxidative stress, and their corresponding pharmacological efficacies significantly depend on an important material processing parameter (i.e., redox radical initiation reaction temperature).

## Conclusions

In conclusions, we show that physicochemical properties of GNGA carriers are strongly affected by redox radical initiation reaction temperatures during polymer synthesis of the carrier material. Increasing reaction temperature maximizes GA grafting at 40 °C. Meanwhile, the increase in overall hydrophilicity of GNGA carriers due to high degree of antioxidant grafting leads to fast polymer degradation and early pilocarpine depletion *in vivo*, which is disadvantageous to achieve extended drug release. In addition, after intracameral pilocarpine administration using optimized GNGA carriers synthesized at 30 °C, we noted the high drug concentrations in aqueous humor of rabbit eyes above therapeutic level for 28 days. This observation is in accordance with levels of reduction in abnormal IOP elevation and corneal aberration in an animal model of experimental glaucoma. Pharmacological efficacy of pilocarpine/GNGA injections in treating glaucomatous injury also suggests the dependence of maintenance of retinal tissue structure and electrophysiological function on grafting amount of GA mediated by redox radical initiation reaction temperature. Biochemical assays reveal that antioxidant-functionalized injectable hydrogels can increase activities in retinal antioxidant defense system, thereby providing the benefits to support a healthy metabolism using antioxidant polymeric drug delivery systems against glaucoma development.

## Methods

### Materials

Type A gelatin (300 Bloom), gallic acid (GA), ascorbic acid, hydrogen peroxide, ammonium molybdate, 2,2′-diphenyl-1-picrylhydrazyl (DPPH), matrix metalloproteinase-2 (MMP-2, EC 3.4.24.24), pilocarpine nitrate, and α-chymotrypsin were purchased from Sigma-Aldrich (St. Louis, MO, USA). *N*-isopropylacrylamide (NIPAAm), from Acros Organics (Geel, Belgium), was purified by recrystallization from n-hexane. Deionized water was filtered using a Milli-Q system (Millipore, Bedford, MA, USA). Balanced salt solution (BSS, pH 7.4) and phosphate-buffered saline (PBS, pH 7.4) were obtained from Alcon (Fort Worth, TX, USA) and Biochrom (Berlin, Germany), respectively. Eagle’s minimum essential medium (MEM) was purchased from Gibco-BRL (Grand Island, NY, USA). Fetal bovine serum (FBS) and the antibiotic/antimycotic (A/A) solution (10,000 U/ml penicillin, 10 mg/ml streptomycin, and 25 μg/ml amphotericin B) were obtained from Biological Industries (Kibbutz Beit Haemek, Israel). All the other chemicals were of reagent grade and used as received without further purification.

### Synthesis of GA-functionalized gelatin-*g*-PNIPAAm (GNGA)

GNGA carrier was synthesized in accordance with a previously established method^6^. In summary, GN copolymers were obtained by using carbodiimide coupling chemistry to attach carboxylic end-capped PNIPAAm onto the aminated gelatin^38^. Specifically, the feed molar ratio of NH_2_ groups in the aminated gelatin to COOH groups in the carboxylic end-capped PNIPAAm was controlled at 0.36 for preparation of GN samples. Later, aqueous GN solution, 1 wt% of GN in deionized water, was used in conjunction with a redox reaction initiator consisting of 0.25 g ascorbic acid and 1 ml of hydrogen peroxide at temperatures of 20, 30, 40, and 50 °C for 90 min. Following the addition of 60 mg of GA, the solutions were allowed to mix thoroughly by gentle agitation for 24 h and dialyzed exhaustively against deionized water to remove unreacted components. The purified product was lyophilized at −50 °C. Here, the GNGA samples obtained by controlling the redox reaction at 20 °C was designated as T20.

### Characterization studies

Physicochemical characterizations of GNGA carriers were performed in accordance with previously established protocols^7^. Briefly, total antioxidant activities and free radical scavenging abilities of GNGA materials were determined by phosphomolybdenum and DPPH assays. While the phosphomolybdenum method is based on the reduction of Mo(VI) to Mo(V) by the antioxidant compounds, the DPPH method is based on the reduction of the stable DPPH radicals by the antioxidant agents^6^. Water content, phase transition temperature, weight loss, and drug encapsulation efficiency were measured to correlate drug delivery performance with grafting amount of GA onto carriers.

### *In vitro* antioxidant activity studies

To evaluate the antioxidant activity of GNGA materials against oxidative stress, the human lens epithelial (HLE-B3; ATCC No: CRL-11421) cell lines were purchased from the American Type Cell Collection (Manassas, VA, USA) and maintained in MEM supplemented with 20% FBS, 2 mM l-glutamine, 1 mM sodium pyruvate, 0.1 mM nonessential amino acids, 1.5 mg/ml sodium bicarbonate, and 1% A/A solution. Intracellular generation of reactive oxygen species (ROS) and overload of calcium were measured by staining the cells with 10 μM 2′,7′-dichlorodihydrofluorescein diacetate (DCFH-DA) (Molecular Probes, Eugene, OR, USA) and 5 μM Fura-2, AM (Molecular Probes) at 37 °C for 1 h, respectively. Detailed procedures for hydrogen peroxide-induced oxidative stress challenge were reported in our previous study^6^. Briefly, HLE-B3 cells with a density of 5 × 10^4^ cells/well were seeded in 24-well plates followed by incubation with 150 μl of sterile GNGA solutions (10% w/v) for 24 h. Then, the cell cultures were further incubated in medium containing 200 μM hydrogen peroxide for 24 h. Fluorescence imaging was acquired with a fluorescence microscope (Axiovert 200 M; Carl Zeiss, Oberkochen, Germany) for ROS (Ex. 488 nm; Em. 525 nm) and calcium (Ex. 340 nm; Em. 510 nm). For comparison purpose, cells exposing to hydrogen peroxide of 0 μM and 200 μM for 24 h following 24 h of incubation in the absence of the polymer carrier materials were designated as Control and HP groups, respectively. To generate pseudocolored images, the acquired images were analyzed using ImageJ software^39^. Furthermore, the fluorescence reading was done with a multimode microplate reader (BioTek Instruments, Winooski, VT, USA) to detect the difference in the fluorescence intensity. All experiments were performed in triplicate. The levels of intracellular ROS and calcium were normalized to the total cell number, analyzed with WST-1 (also see the Supplementary Information).

### Animals

All animal procedures were approved by the Institutional Review Board of Chang Gung University (IACUC approval number: CGU13–024) and were conducted in accordance with the ARVO Statement for the Use of Animals in Ophthalmic and Vision Research. In this study, adult New Zealand white rabbits (National Laboratory Animal Breeding and Research Center, Taipei, Taiwan, ROC), weighing 3.0–3.5 kg and 16–20 weeks of age, were used for *in vivo* studies. Animals were healthy and free of clinically observable ocular surface disease. Surgical operation was performed in the single eye of animals, with the normal fellow eye. During surgery and follow-up, the rabbits were anesthetized intramuscularly with 2.5 mg/kg body weight of tiletamine hydrochloride/zolazepam hydrochloride mixture (Zoletil; Virbac, Carros, France) and 1 mg/kg body weight of xylazine hydrochloride (Rompun; Bayer, Leverkusen, Germany).

### *In vivo* biocompatibility studies

Fifteen rabbits were used for *in vivo* biocompatibility studies. In the four test groups (T20, T30, T40, and T50) of animals (3 rabbits/group), the rabbits received 50 μl intracameral GNGA injections. The remaining 3 rabbits received buffer solution without GNGA polymer and served as a control group. To determine the tissue responses to materials, ophthalmic evaluations were performed after 28 days of intracameral polymer injection. Corneal endothelial cell morphology and density in rabbit eyes were observed and measured using specular microscopy (Topcon Optical, Tokyo, Japan)^40^. Each data point represents an average of three independent observations. After sacrifice with CO_2_, the rabbit corneas were excised and processed for histological examinations^41^. Tissue samples were fixed in 4% paraformaldehyde in PBS, dehydrated in a graded series of ethanol solutions, embedded in paraffin, and cut into 5 μm sections. Thin sections were stained with hematoxylin and eosin (H&E) and examined under light microscope (Carl Zeiss).

On the other hand, the aqueous humor specimens from each rabbit eye was immediately aspirated using a 30-gauge needle without touching the iris, lens, and corneal endothelium. The concentrations of IL-6 in aqueous humor were determined by using a sandwich enzyme-linked immunosorbent assay (ELISA) kit (MyBioSource, San Diego, CA, USA) according to the manufacturer’s instructions. For cytokine bioassays, photometric readings at 450 nm were measured using the Spectrophotometer (ThermoLabsystems, Vantaa, Finland). Results were expressed as pg/ml. All experiments were conducted in triplicate.

### *In vivo* drug release studies

Experimental glaucoma model, induced by injection of 0.1 mg/ml of α-chymotrypsin into the posterior chamber of rabbit eye, was established according to our previously published methods^5^. The animals were considered to be glaucomatous (GL) when the IOP was higher than 20 mmHg in the eye following 4 weeks of α-chymotrypsin injection. To evaluate *in vivo* drug release at specific time intervals, forty-eight white rabbits were randomized into four experimental groups receiving intracameral injections of 50 μl of a mixture containing pilocarpine nitrate (2% w/v) and GNGA solutions (10% w/v) from T20, T30, T40, and T50 groups (12 rabbits/group). For *in vivo* drug release studies, three rabbits from each group were euthanized with CO_2_ at postoperative days 3, 7, 14, and 28. The aqueous humor from each rabbit eye was aspirated using a 30-gauge needle. The concentrations of released pilocarpine nitrate in aqueous humor specimens were analyzed by high performance liquid chromatography (HPLC) according to our previously published methods^42^. All experiments were conducted in triplicate.

### Glaucoma therapy studies

In a separate animal study, an additional thirty-six glaucomatous rabbits following 4 weeks of α-chymotrypsin injection were used for examination of antiglaucoma efficacy. For comparison, 6 glaucomatous rabbits in GL groups were included in the design of the study. In the four test groups (T20, T30, T40, and T50) of animals (6 rabbits/group), the glaucomatous rabbits received intracameral injections of 50 μl of a mixture containing pilocarpine nitrate (2% w/v) and GNGA solutions (10% w/v). Without treatment with any polymers and drugs, the remaining 6 rabbits with experimental glaucoma served as a control group (Ctrl) and examined during 4 weeks of follow-up.

The IOP of bilateral eyes were measured at predetermined time intervals using a Schiotz tonometer (AMANN Ophthalmic Instruments, Liptingen, Germany), calibrated according to the manufacturer’s instructions^43^. For each IOP determination, five readings were taken on each eye, alternating the left and right eyes, and the mean was calculated. The IOP values of the contralateral normal eyes were used as baseline readings. Data were expressed as the difference from baseline values at each time point.

Corneal topographic profiles were studied by using the Medmont E300 Corneal Topographer (Medmont Pty Ltd., Melbourne, Australia) at 28 days postoperatively. Prior to data acquisition, calibrations were performed on the corneal topographer according to manufacturer’s recommendations (±0.01 mm at 4 calibration surfaces)^44^. Mean keratometric (K) value, representing average measurement of the corneal curvature at central 3 mm zone, was recorded from topographic map^45^. Each data point is an average of three independent observations.

The electroretinogram (ERG) was also studied with flash stimulation in rabbits. The examinations were conducted according to the International Society for Clinical Electrophysiology of Vision (ISCEV) standards^46^. For ERG measurements, the animals were anesthetized to allow for recording without electrical noise from muscle activity. The rabbit eye was tested after maximal pupil dilation with topical 1% cyclopentolate hydrochloride ophthalmic solution (Alcon). Following at least 30 min of dark adaptation, an Ag/AgCl electrode (Biopac Systems, Santa Barbara, CA, USA) was applied on the topically anesthetized cornea, a reference electrode on the ear, and a subcutaneous ground electrode on the neck of the rabbit. The ERGs were recorded from each eye, and 10 responses to flashes of white light (4 ms, 1 Hz) from a photic stimulator set at maximum brightness (90 cd s/m^2^ without a filter) were amplified and filtered. Electrophysiological responses were averaged for each run, and the mean of the five independent runs was used for subsequent analysis of the peak amplitudes of the a- and b-waves of the ERG. The a-wave was measured as the difference in amplitude between the recording at onset and the trough of the negative deflection and the b-wave was measured as the difference in amplitude between the trough of the a-wave to the peak of the b-wave. Each data point is an average of three independent measurements.

At the end of the study (i.e., 28 days), the animals were euthanized with CO_2_ and the eyes were enucleated for histological examination^47^. Tissue samples were fixed with 4% paraformaldehyde. Following removal of anterior segments, the posterior eyecups were mounted onto precooled chucks in embedding medium (OCT Tissue-Tek; Sakura Finetek, Torrance, CA, USA) and frozen at −70 °C. Frozen specimens were prepared with the use of a cryostat into 5 μm sections at −20 °C. Cryosections were cut along the vertical meridian of the eye through the optic nerve (superior-inferior). Thin sections were further stained with H&E and observed under a calibrated optical microscope (Nikon, Melville, NY, USA) to determine the total retinal thickness (from inner to outer limiting membrane)^48^. The thickness was measured in three adjacent areas within 1 mm distance to the optic nerve center. Each data point is an average of four independent measurements.

Biochemical assays, including superoxide dismutase (SOD), catalase (CAT), and glutathione peroxidase (GPx) activities, and glutathione (GSH) levels, were also measured in accordance with a published method^22^. Assay preparation involved in the homogenization of rabbit retinal tissues in aqueous buffers. In SOD assay, epinephrine underwent autoxidation rapidly at pH 10.0 and produced adrenochrome (i.e., a pink-colored product) that absorbs at 480 nm and can be quantitated spectrophotometrically. The amount of SOD required to produce 50% inhibition of epinephrine autoxidation was defined as one unit of enzyme activity. Total SOD activity was expressed as U/mg of protein. In CAT assay, homogenate (200 μl) was mixed with 25 μl of 20% Triton X-100 and incubated for 5 min at 4 °C. After 1 min of centrifugation, 50 μl of supernatant was diluted with 900 μl of 50 mM PBS followed by addition of 50 μl of 200 mM H_2_O_2_. Using an UV-vis spectrophotometer (Thermo Scientific, Waltham, MA, USA), absorbance at 240 nm was measured for 100 s every 10 s. CAT activity was calculated with the extinction coefficient of H_2_O_2_ at 240 nm (0.04 mM^−1^·cm^−1^), and expressed as nmol H_2_O_2_/min/mg of protein. In GPx assay, homogenate was mixed with EDTA, NaN_3_, NADPH, GSH, and glutathione reductase in potassium phosphate buffer (50 mM, pH 7.4). Enzyme source was added to the above reaction mixture and incubated at 37 °C for 5 min before initiation of the reaction using 100 μl of 2 mM H_2_O_2_. The absorbance was spectrophotometrically read at 340 nm. GPx activity was calculated with the extinction coefficient of NADPH at 340 nm (6.22 mM^−1^·cm^−1^), and expressed as nmol/mg of protein In GSH assay, homogenate was mixed with 50% TCA and 1 mM EDTA for 5 min at 4 °C. The samples were centrifuged and mixed with 0.25 mg/ml 5,5′-dithio-bis(2-nitrobenzoic acid). The absorbance was spectrophotometrically read at 412 nm. GSH level was expressed as nmol/mg of protein.

### Statistical analyses

Results were expressed as mean ± standard deviation (SD). Comparative studies of means were performed using a one-way analysis of variance (ANOVA) followed by a Newman-Keuls post hoc test. Significance was accepted with *P* < 0.05.

## Additional Information

**How to cite this article**: Chou, S.-F. *et al*. In Vivo Pharmacological Evaluations of Pilocarpine-Loaded Antioxidant-Functionalized Biodegradable Thermogels in Glaucomatous Rabbits. *Sci. Rep.* **7**, 42344; doi: 10.1038/srep42344 (2017).

**Publisher's note:** Springer Nature remains neutral with regard to jurisdictional claims in published maps and institutional affiliations.

## Supplementary Material

Supplementary Information

## Figures and Tables

**Figure 1:**
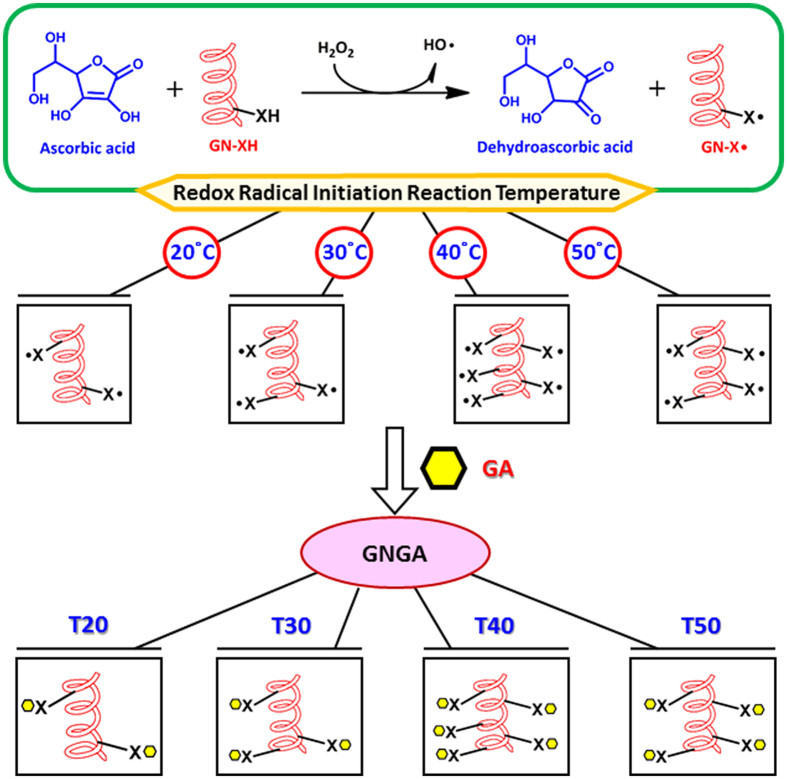
Schematic representation of functionalization of GN with GA molecules. In the presence of hydrogen peroxide/ascorbic acid redox pair as an initiator, GN copolymers containing varying amounts of radical species are obtained at different redox radical initiation reaction temperatures for further grafting with antioxidant GA.

**Table 1:** Characterization studies of various GNGA samples.

Sample code	Total antioxidant activity^a^ (mg/g polymer)	Inhibition of DPPH radical^b^ (%)	Water content^c^ (%)	LCST^d^ (°C)	Weight loss^e^ (%)	Drug encapsulation efficiency^f^ (%)
T20	0.1 ± 0.04	23.7 ± 4.9	54.2 ± 1.5	26.9 ± 0.5	63.4 ± 1.1	62.8 ± 1.6
T30	0.4 ± 0.08^g^	45.6 ± 2.3^g^	65.5 ± 2.1^g^	29.8 ± 0.3^g^	67.3 ± 1.4^g^	72.5 ± 1.9^g^
T40	1.5 ± 0.07^g^	84.7 ± 3.4^g^	78.1 ± 1.8^g^	32.5 ± 0.3^g^	72.6 ± 1.3^g^	74.1 ± 2.0^g^
T50	1.3 ± 0.11^g^	67.3 ± 1.8^g^	73.2 ± 1.4^g^	31.3 ± 0.2^g^	69.8 ± 0.9^g^	72.8 ± 1.3^g^

^a^Determination of total antioxidant activity by phosphomolybdenum assay. Data are expressed as mean ± standard deviation (*n* = 6).

^b^Determination of free radical scavenging activity by 2,2′-diphenyl-1-picrylhydrazyl (DPPH) method. Data are expressed as mean ± standard deviation (*n* = 6).

^c^Determination of water content by gravimetric method. Data are expressed as mean ± standard deviation (*n* = 5).

^d^Determination of lower critical solution temperature (LCST) by differential scanning calorimetry (DSC). Data are expressed as mean ± standard deviation (*n* = 5).

^e^Determination of weight loss by gravimetric method after 28 days of incubation at 34 °C in the presence of matrix metalloproteinase-2 (MMP-2). Data are expressed as mean ± standard deviation (*n* = 6).

^f^Determination of drug encapsulation efficiency by high-performance liquid chromatography (HPLC). Data are expressed as mean ± standard deviation (*n* = 4).

^g^Significant difference as compared to the T20 groups (*P* < 0.05).
