# Supplementary material for: In vivo Pharmacological Evaluations of Pilocarpine-Loaded Antioxidant-Functionalized Biodegradable Thermogels in Glaucomatous Rabbits
Source: Sci Rep. 2017 Feb 10;7:42344. doi: 10.1038/srep42344 (PMC5301226; doi:10.1038/srep42344)
Supplement: Supplementary Information [file srep42344-s1.pdf]

Supplementary Information

In Vivo Pharmacological Evaluations of Pilocarpine-Loaded Antioxidant-Functionalized Biodegradable Thermogels in Glaucomatous Rabbits

Shih-Feng Chou[1,†], Li-Jyuan Luo[2,†], and Jui-Yang Lai[3,4,5,6,7,*]

[1]Department of Mechanical Engineering, University of Texas at Tyler, Tyler TX, 75799, USA
[2]Department of Chemical and Materials Engineering, Chang Gung University, Taoyuan 33302, Taiwan, ROC
[3]Institute of Biochemical and Biomedical Engineering, Chang Gung University, Taoyuan 33302, Taiwan, ROC
[4]Biomedical Engineering Research Center, Chang Gung University, Taoyuan 33302, Taiwan, ROC
[5]Center for Tissue Engineering, Chang Gung Memorial Hospital, Taoyuan 33305, Taiwan, ROC
[6]Department of Ophthalmology, Chang Gung Memorial Hospital, Taoyuan 33305, Taiwan, ROC
[7]Department of Materials Engineering, Ming Chi University of Technology, New Taipei City 24301, Taiwan, ROC

[*]jylai@mail.cgu.edu.tw
[†]these authors contributed equally to this work

In vitro antioxidant activity studies

HLE-B3 cells with a density of 5×10^4 cells/well were seeded in 24-well plates followed by incubation with 150 μl of sterile GNGA solutions (10% w/v) for 24 h. Then, the cell cultures were further incubated in medium containing 200 μM hydrogen peroxide for 24 h. The qualitative and quantitative assays were performed to measure the cell viability. Cell morphology was observed by phase-contrast microscopy (Nikon, Melville, NY, USA) [#1]. Furthermore, HLE-B3 cells were counted by using the cell proliferation reagent WST-1 (Roche Diagnostics, Indianapolis, IN, USA) assay. The WST-1 assay is based on the cleavage of the tetrazolium salt WST-1 (4-[3-(4-iodophenyl)-2-(4-nitrophenyl)-2H-5-tetrazolio]-1,3-benzene disulfonate) to a colored formazan by mitochondrial dehydrogenases in viable cells [#2]. The amount of formazan product is proportional to the number of metabolically active cells. For staining, 100 μl of WST-1 reagent was added to the cultures, and incubated for 4 h at 37°C in a CO_2 incubator. The optical density (OD) value at 450 nm was recorded using the Multiskan Spectrum Microplate Spectrophotometer (ThermoLabsystems, Vantaa, Finland). All experiments were performed in quadruplicate.

Representative images of HLE-B3 cells photographed after 2 days of cultivation are shown in Figure S2a. In the control groups, the cells without pretreatment with GNGA materials and exposure to hydrogen peroxide appeared healthy and exhibited typical lens epithelial

morphological characteristics. By contrast, the cells in the HP groups showed shrinkage and partial detachment from the culture substrate, indicating the generation of hydrogen peroxide-related oxidative stress damage. In the T20, T30, T40, and T50 groups, the loss of cellular morphology as a result of oxidative stress was prevented by the pretreatment with GNGA samples. Results of quantitative analysis of cell viability are also shown in Figure S2b. The order of metabolic activity followed T40 > T50 > T30 > T20 > HP, suggesting that the pretreatment with GNGA materials can increase the cell survival rate. The polymer samples with high grafting amount of GA are more effective in alleviating the hydrogen peroxide-induced oxidative injury by enhancing the antioxidant action.

References

[#1] Lai, J. Y. & Tu, I. H. Adhesion, phenotypic expression, and biosynthetic capacity of corneal keratocytes on surfaces coated with hyaluronic acid of different molecular weights. *Acta Biomater.* **8,** 1068–1079 (2012).

[#2] Chou, S. F., Luo, L. J. & Lai, J. Y. Gallic acid grafting effect on delivery performance and antiglaucoma efficacy of antioxidant-functionalized intracameral pilocarpine carriers. *Acta Biomater.* **38,** 116–128 (2016).

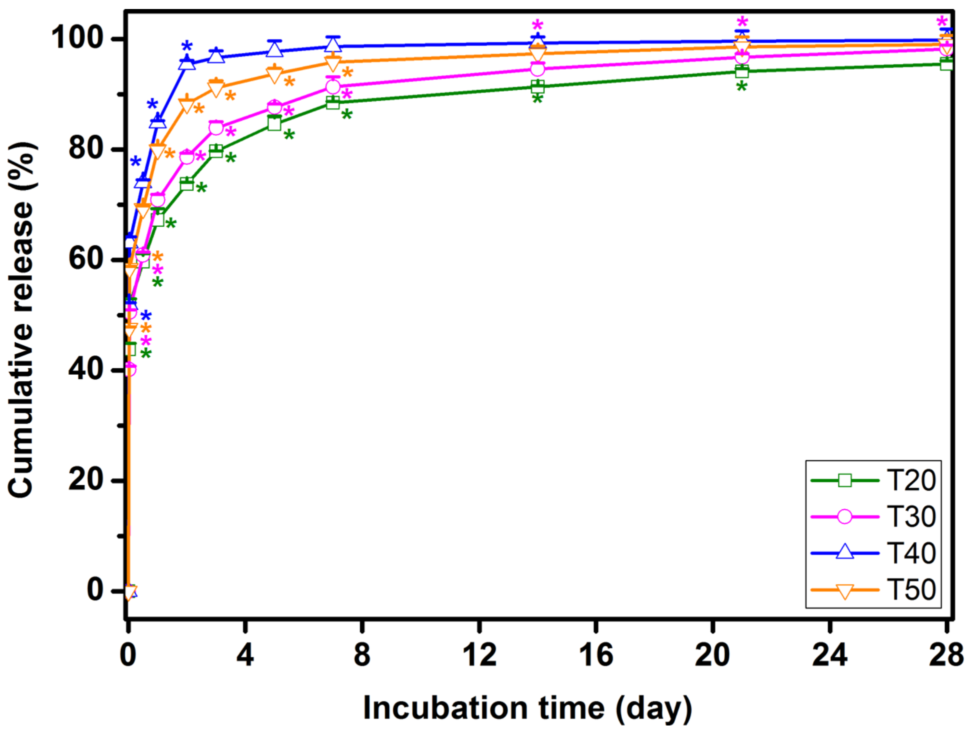

Figure S1. In vitro drug release studies. Cumulative release percentage of pilocarpine released from various GNGA samples T20, T30, T40, and T50 at 34°C in BSS containing MMP-2. An asterisk indicates statistically significant differences (**P* < 0.05; *n* = 4) for the mean value of the cumulative release percentage compared to the value at the previous time point.

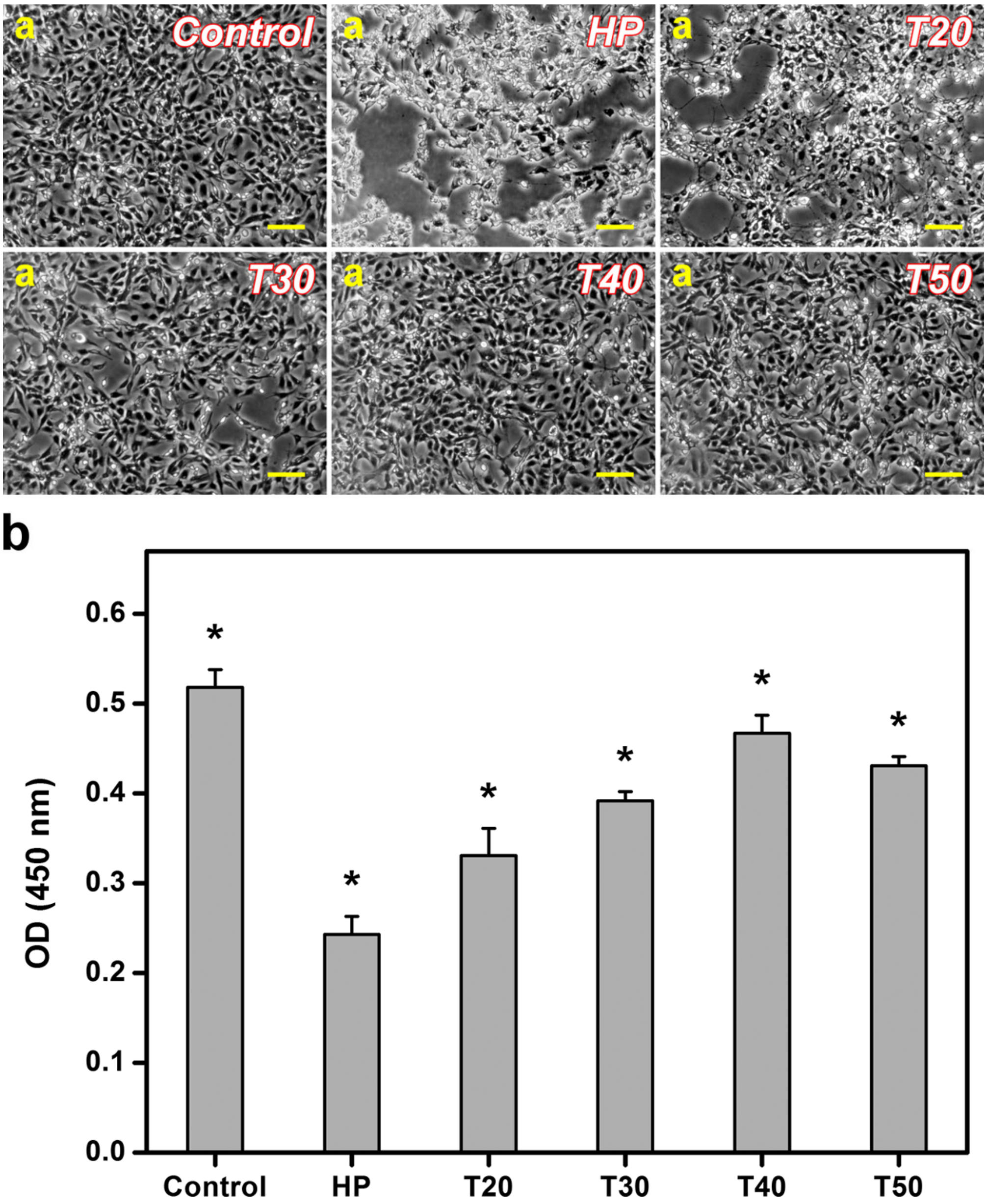

Figure S2. In vitro antioxidant activity studies. Effect of polymer carrier materials on H_2O_2-induced cell viability. (**a**) Representative phase-contrast micrographs of the HLE-B3 cells after incubation with various GNGA samples T20, T30, T40, and T50 for 24 h and further exposure to H_2O_2 for 24 h. The cells exposed to 0 (Control group) or 200 (HP group) μM H_2O_2 for 24 h following 24 h of incubation in the absence of the polymers are used for comparison. Scale bars: 100 μm. (**b**) Cell viability was measured by the WST-1 assay. Results of metabolic activity were expressed as the OD value at 450 nm. Values are mean ± standard deviation (n = 4). *$P < 0.05$ vs all groups.

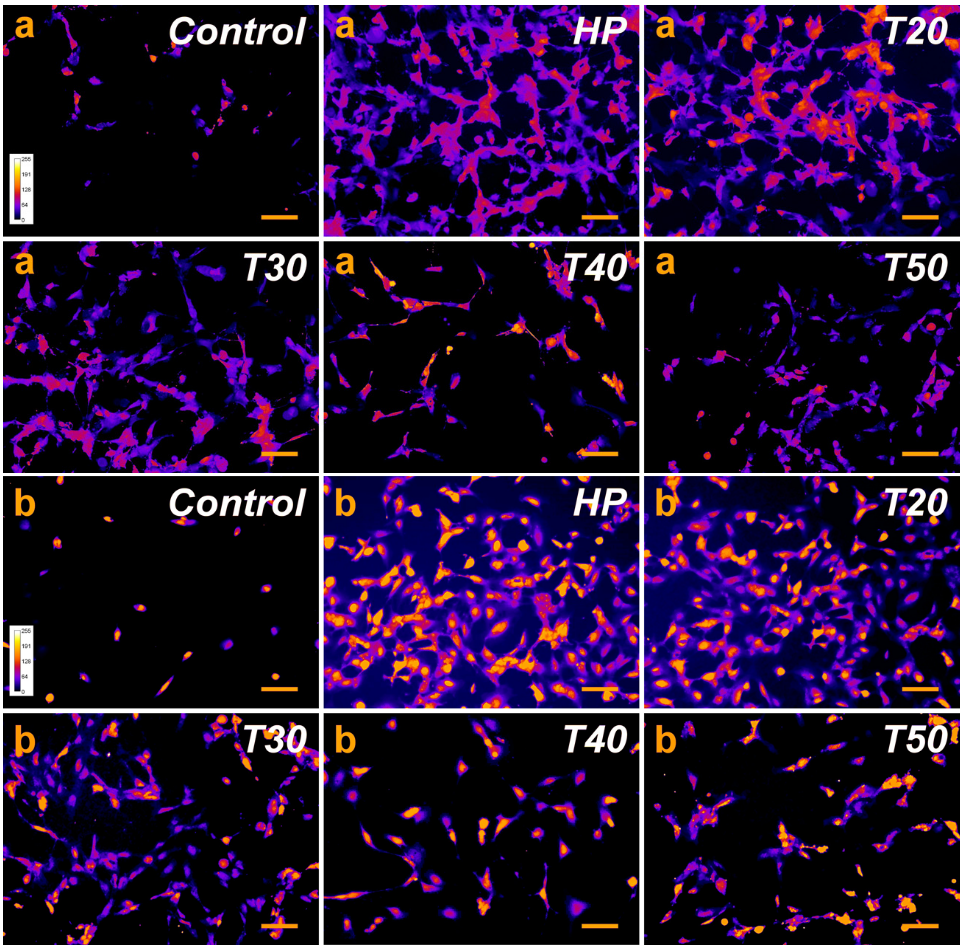

Figure S3. In vitro antioxidant activity studies. Effect of polymer carrier materials on H_2O_2-induced intracellular (**a**) ROS and (**b**) calcium. Pseudocolored images of the HLE-B3 cells after incubation with various GNGA samples T20, T30, T40, and T50 for 24 h and further exposure to H_2O_2 for 24 h. The cells exposed to 0 (Control group) or 200 (HP group) μM H_2O_2 for 24 h following 24 h of incubation in the absence of the polymers are used for comparison. Yellow/red and blue colors show the regions of high and low oxidative stress, respectively.

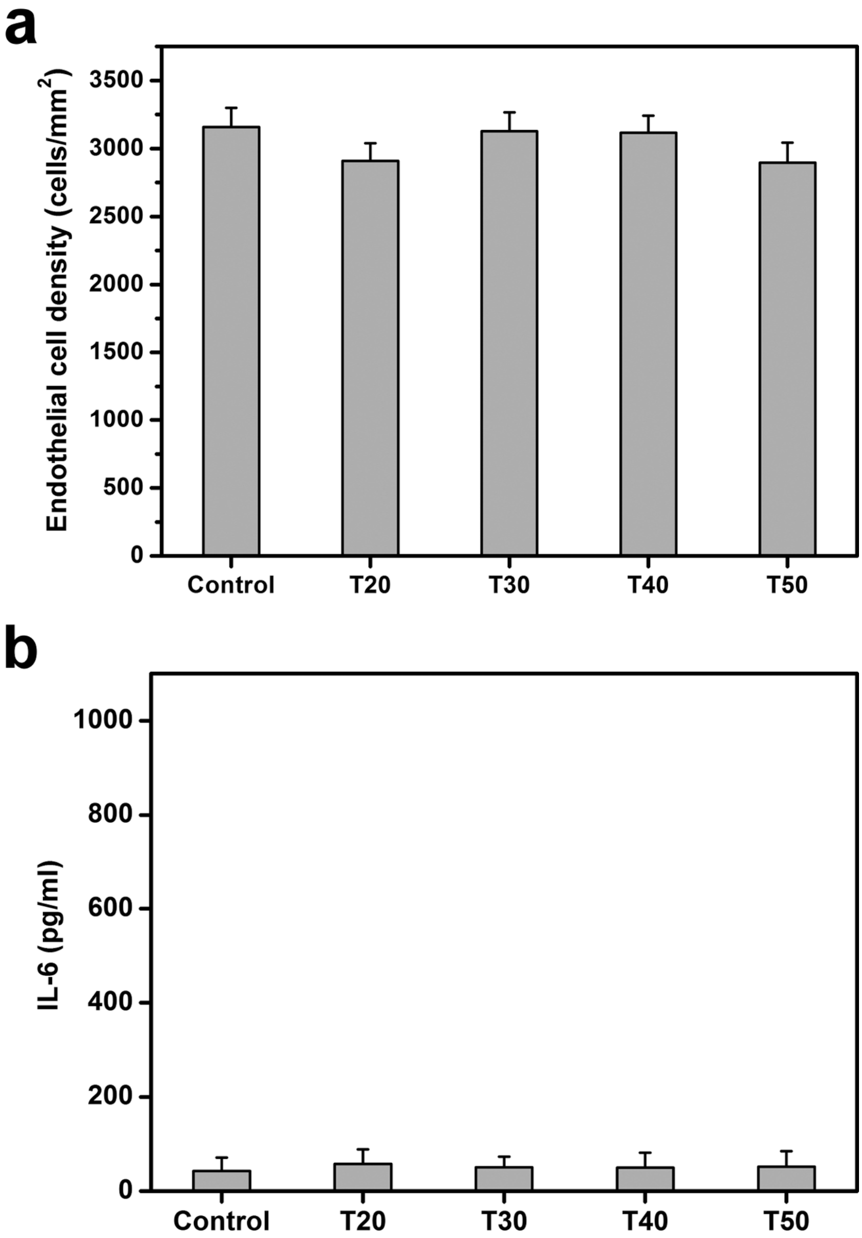

Figure S4. In vivo biocompatibility studies. (**a**) Specular microscopy measurements of corneal endothelial cell density. Control group: sham operation (no material). Values are mean ± standard deviation (*n* = 3). (**b**) Level of IL-6 protein in aqueous humor from GNGA-implanted rabbits. Control group: sham operation (no material). Values are mean ± standard deviation (*n* = 3).

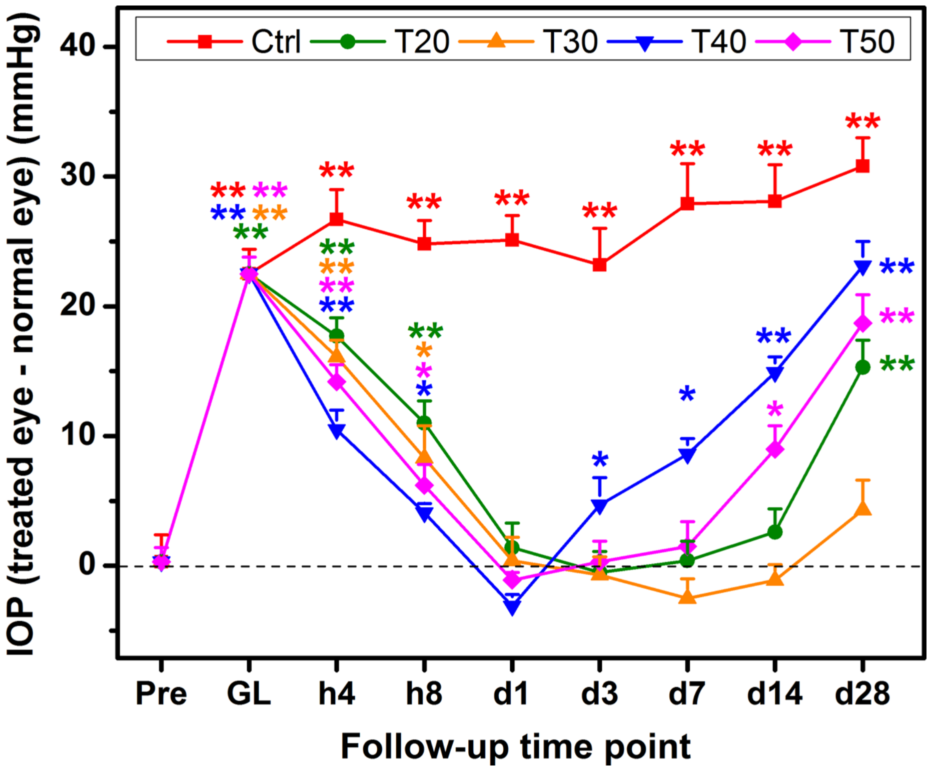

Figure S5. Evaluation of antiglaucoma efficacy. Measurements of IOP after intracameral injection of various pilocarpine-containing GNGA polymer solutions (T20, T30, T40, and T50) in rabbits with glaucoma (GL). Glaucomatous animals receiving no polymer and drug serve as control groups (Ctrl). Asterisks indicate statistically significant differences (*$P < 0.05$; **$P < 0.005$; $n = 6$) as compared with the baseline IOP values. Follow-up time point: preoperation (Pre); hour (h); day (d).

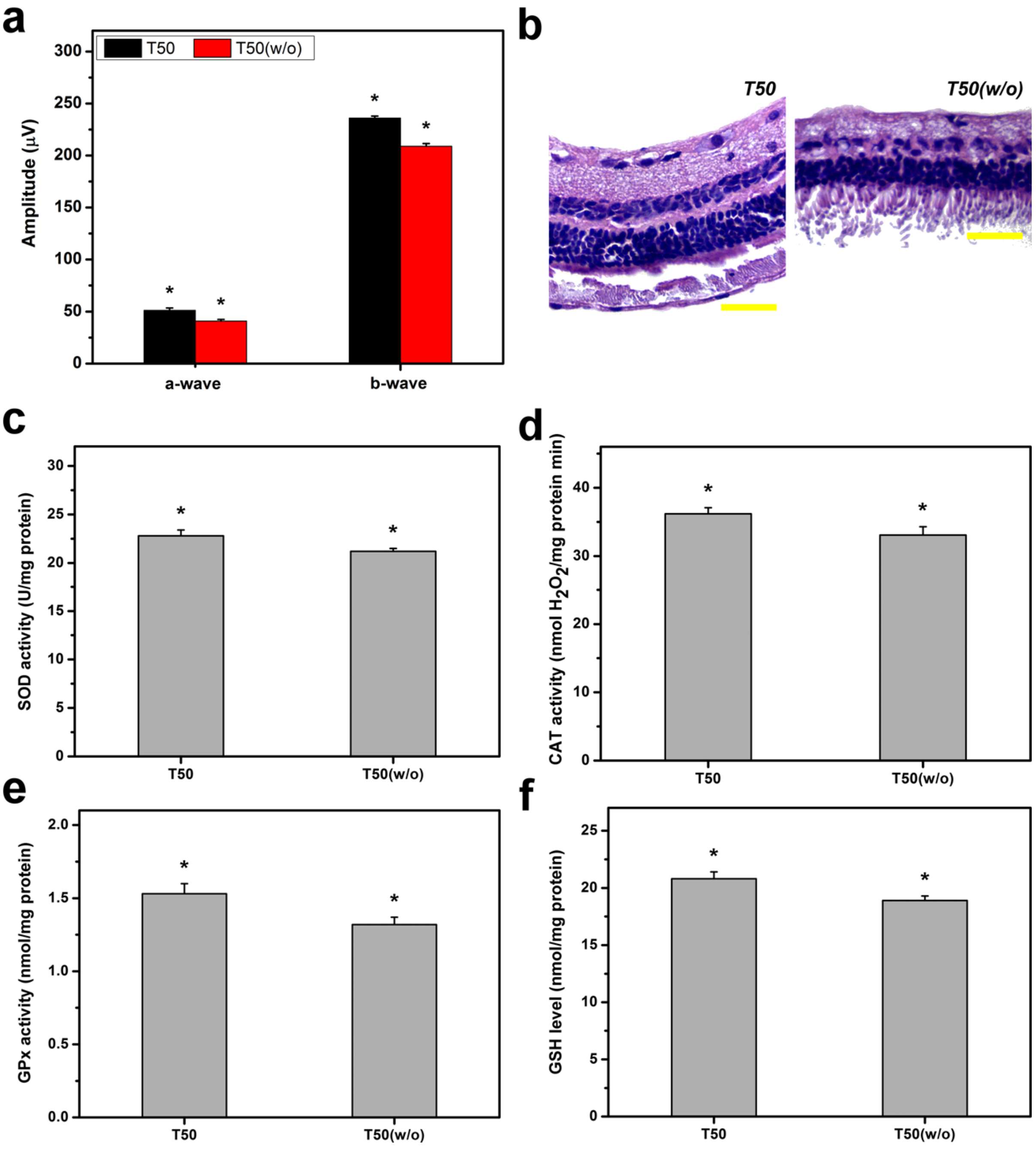

Figure S6. Glaucoma therapy studies. (**a**) ERG waveform amplitudes, (**b**) retinal histological images, (**c**) retinal SOD activity, (**d**) retinal CAT activity, (**e**) retinal GPx activity, and (**f**) retinal GSH level of glaucomatous rabbits 7 days after intracameral injection of mixtures of pilocarpine and GNGA (T50 groups) and GNGA alone (T50(w/o) groups). Scale bars: 50 μm. Values are mean ± standard deviation (n = 6). *P < 0.05 vs all groups.
